# Changing Pattern of Pediatric Endocrinology Referrals Over Two Decades: A Study From a Tertiary Center in Western India

**DOI:** 10.7759/cureus.91326

**Published:** 2025-08-31

**Authors:** Vaman Khadilkar, Karishma Bhade, Sonali Wagle, Anuradha Khadilkar

**Affiliations:** 1 Growth and Pediatric Endocrinology, Hirabai Cowasji Jehangir Medical Research Institute, Pune, IND; 2 Health Sciences, Savitribai Phule Pune University, Pune, IND

**Keywords:** discrepancy, disorders of puberty, icped, obesity, pediatric endocrinology, referrals, short stature

## Abstract

Introduction

Understanding the pediatric endocrinology referral pattern is important for primary care clinicians and pediatric endocrinologists to optimize patient care, facilitate continuous medical education, and upgrade resources. This study analyzed the pattern of these referrals over a span of one year each, two decades apart, and the difference between the referral reasons and final diagnoses.

Methods

A retrospective analysis was conducted on demographic details and referral reasons to a pediatric endocrine clinic in a tertiary care hospital in Western India at two time intervals two decades apart (2002-2003 and 2022-2023). The referral reasons were categorized into 14 classes as per the International Classification of Pediatric Endocrinology Diagnoses (ICPED) 2016.

Results

Data of 2595 patients (920 (35.4%) from 2002-2003 and 1675 (64.5%) from 2022-2023) were studied. The most common reason for referral was short stature without gender bias. Disorders of puberty and obesity were the second and third most common reasons for referral. There was almost a two-fold rise in the total referrals over two decades, with a significant rise in females referred for short stature and disorders of puberty. There was a discrepancy between the final diagnosis and referral reason, predominantly in patients referred for micropenis, gynecomastia, and obesity.

Conclusion

We report pediatric endocrine referral patterns two decades apart, revealing a shift in the number of referrals with minor shifts in the referral reasons. A gap exists in recognizing symptoms and possible causes at the primary care level. These findings highlight the need for focused medical education and awareness among primary care clinicians.

## Introduction

Disorders of hormonal imbalance and stature are common causes of concern for parents and children and, hence, are referred to pediatric endocrine services [1-3]. As demographic changes lead to variation in the disease frequency and patterns, it is necessary to study and document the changing disease spectrum over time that gets referred to any specialty unit; however, there is a scarcity of data on the referral patterns of pediatric endocrine disorders in Indian literature. 

Over the past few decades, the incidence of precocious puberty and type 1 diabetes have been reported with increasing frequency. Further, as India goes through the phase of economic and nutritional transition, obesity is also on the rise [4]. The initial contact for a child's health issues typically begins with a general pediatrician who plays a critical role in the primary care of a child, including identifying and recommending specialty consultations as needed. Effectively addressing these concerns and ensuring appropriate referrals to a pediatric endocrinologist when necessary are essential for delivering timely and effective treatment [5]. Understanding the pattern of referral aids in organizing and prioritizing outpatient clinics of tertiary referral centers. 

Among all the endocrine disorders, the referral pattern and etiology of short children have been well documented in Indian studies; however, there are very few Indian or global studies describing the pattern of overall pediatric endocrine referral [6,7]. In 2020, Bellotto et al. conducted a retrospective study analyzing referral patterns in pediatric endocrinology over a six-year period at a single tertiary center in Italy. This study underscores the importance of improving primary care education on appropriate referral timing, prioritization, and distinguishing between normal variations and pathological conditions to optimize healthcare resource utilization [8].

The purpose of the present study was to retrospectively analyze changing patterns of pediatric endocrine referral trends to a tertiary-level center in Western India two decades apart (2002-2003 vs. 2022-2023) and to compare the discrepancy between reasons for referral and the final diagnosis.

This article was previously posted to the medRxiv preprint server.

## Materials and methods

Study design

This is a retrospective, observational study where we analyzed data on patients referred to the pediatric endocrine outpatient unit of our tertiary care hospital in Western India at two time intervals 20 years apart. The first dataset is from January 2002 to December 2003, and the second dataset is from January 2022 to December 2023.

Participants

We included all the children and adolescents aged 0 to 21 years referred by primary health care providers to a tertiary-level pediatric endocrine clinic were included in the study. The exclusion criteria included children and adolescents who lost to follow-up before the final diagnosis was made. 

The deidentified data were procured from the treating consultant's electronic records. Information regarding the reason for referral, sex, and date of birth was retrieved at both time intervals. Patients were categorized based on the referral reasons into 14 classes as per the International Classification of Pediatric Endocrinology Diagnoses (ICPED) 2016 [9]. These classes are short stature, tall stature, puberty, sex development and gender, obesity, pituitary gland, hypothalamus and central nervous system, thyroid gland, adrenal glands, testes and male reproductive tract, ovaries and female reproductive tract, glucose and lipid metabolism, calcium and phosphate metabolism, salt and water regulation, and syndrome with endocrine features. These main classes were further sub-categorized as per ICPED 2016. The final diagnosis was made based on the clinical history and examination, anthropometric parameters, and investigations. 

Statistical analysis

The electronic records were exported to an Excel sheet (Microsoft, Redmond, Washington). Analysis was performed with SPSS version 29 (IBM Inc., Armonk, New York). Data are presented as frequencies and percentages. The differences in the proportions of referral patterns over two decades among males and females were calculated using the z-test for proportion; a p-value <0.001 was considered significant. The Institutional Ethics Committee granted a waiver to analyze data that had been appropriately deidentified to ensure privacy and confidentiality.

## Results

**Figure 1:**
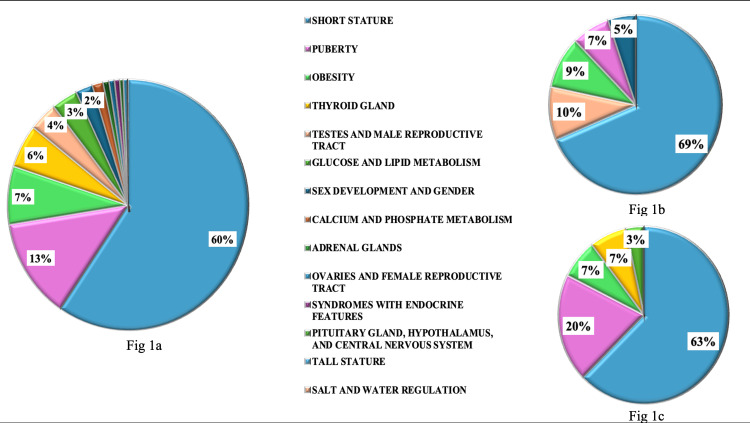
Distribution of referral categories as per proportions in total patients (a), males (b) and females (c) The five most common referral categories are short stature, disorders of puberty, obesity, disorders of the thyroid gland, and disorders of the testes and male reproductive tract. Short stature is the most common reason for referral in both males and females, followed by disorders of the testes and male reproductive tract in males and disorders of puberty in females.

Data on a total of 2595 patients (1675 (64.5%) from 2022-2023 and 920 (35.4%) from 2002-2003) were analyzed. Out of 1675 patients in 2022-2023, 770 (45.9%) were males (median age 11.1 years, IQR- 7.5-13.7) and 905 (54%) were females (median age 10.4 years, IQR- 7.2-13.5). The most common five referral categories were short stature (n=999, 59.6%), puberty (n=217, 13%), obesity (n=125, 7.5%), thyroid gland (n=93, 5.6%), and testes and male reproductive tract (n=64, 3.8%) (Figure 1a). Sex-wise distribution of the five most common main categories is shown in Figure 1b,c. Short stature was the most common reason for referral in both males (59.7%, n=460) and females (n=539, 59.6%). Disorders of the testes and male reproductive tract (8.3%, n=64) and disorders of puberty (18.7%, n=169) were the second most common categories in males and females, respectively. This was followed by obesity (8.2%, n=63), disorders of puberty (6.2%, n=48), and disorders of sex development and gender (4.5%, n=35) in males and obesity (6.9%, n=62), disorders of thyroid gland (6.7%, n=61), and disorders of glucose and lipid metabolism (3.2%, n=29) in females.

Subcategories of the five most common classes 

Short Stature

**Figure 2:**
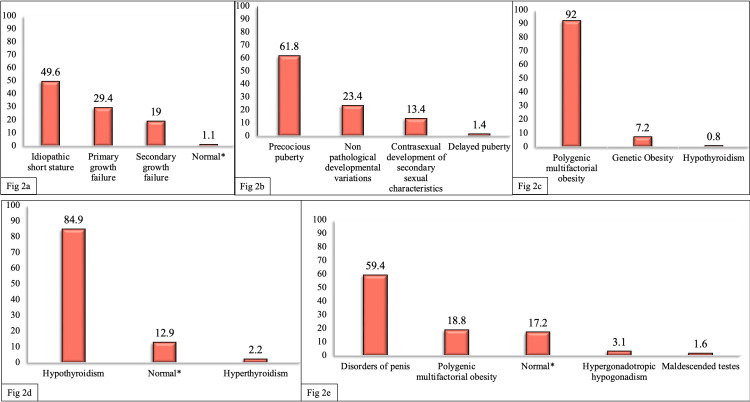
Frequency distribution of subcategories in each of the five most common referral categories * Denotes the subjects who were referred for a particular disorder but found to have no pathological condition and were normal a) Short stature - The most common cause of Short Stature was idiopathic short stature (familial idiopathic short stature: 22.5%, Non-familial idiopathic short stature: 14.5%, constitutional delay in growth and puberty: 11.3%, delayed puberty: 1.3%) b) Puberty - The most common cause of disorders of puberty was precocious puberty c) Obesity - The most common cause of obesity was polygenic multifactorial obesity d) Thyroid gland - The most common cause of disorders of the thyroid gland was hypothyroidism. The findings of 12.9% patients referred for suspected disorders were normal e) Testes and male reproductive tract - The most common cause of disorders of the testes and male reproductive tract was disorders of the penis. 17.2% patients referred with suspected pathology were normal

Among 999 patients with short stature, 49.6% (n=495) were diagnosed to have idiopathic short stature (familial ISS (FSS): 22.5% (n=111), non-familial ISS: 14.5% (n=72), constitutional delay in growth and puberty (CDGP): 11.3% (n=56), delayed puberty: 1.3% (n=6)), 29.4% (n=294) had primary growth failure (small for gestational age with failure to catch up growth: 17.8% (n=52), syndromic short stature: 6.1% (n=18), skeletal dysplasia: 5.5% (n=16)), 19% (n=190) had secondary growth failure (Endocrine causes: 8.3% (n=16), nutritional cause: 6.5% (n=123), chronic illness: 3% (n=6), other skeletal defects: 1.1% (n=2)) and 1.1 % (n=11) had normal stature (Figure 2a).

Puberty

Among 217 patients referred for disorders of puberty, 61.8% (n=134) had precocious puberty, 23.4% (n=50) had non-pathological developmental variations, 13.4% (n=29) had contrasexual development of secondary sexual characteristics in which all the patients were males with puberty gynecomastia, and 1.4% (n=3) had delayed puberty (Figure 2b).

Obesity

Out of 125 patients referred for obesity, 92% (n=115) had polygenic, multifactorial obesity, 7.2% (n=9) had genetic obesity, and 0.8% (n=1) had hypothyroidism (Figure 2c).

Thyroid Gland

Out of 93 patients with disorders of the thyroid gland, 84.9% (n=79) had hypothyroidism, 12.9% (n=12) had no clinical or biochemical pathology and hence labelled normal, and 2.2 % (n=2) had hyperthyroidism (Figure 2d)

Testes and Male Reproductive Tract

Sixty-three males were referred for disorders of the testes and male reproductive tract. Out of these, 78.2% (n=47) had disorders of the penis, 17.4% (n=11) had normal variants, 3.1% (n=2) had hypergonadotropic hypogonadism, and 1.6% (n=1) had maldescended testis (Figure 2e)

Change in the pattern of referrals over two decades 

The total number of referrals and outpatient load had increased from 2002-2003 to 2022-2023 by 82% (n=755). In males, the number increased by 54% (n=270) and in females it increased by 115% (n=485). The referrals for obesity were significantly decreased in males. The proportion of overall referrals for short stature and puberty were increased, but decreased for obesity, disorders of the ovaries and female reproductive tract and the thyroid gland (Table 1).

**Table 1:** Change in the pattern of referral categories over 2 decades in total patients, males and females Values are in n (%), comparison between two groups was done using z test for proportion

	Total			Males			Females		
	2002-03	2022-23	p-value	2002-03	2022-23	p-value	2002-03	2022-23	p-value
Referral categories	n=920	n=1675		n=500	n=770		n=420	n=905	
Short stature	415 (45.1%)	999 (59.6%)	<0.001	222 (44.4%)	460 (59.7%)	0.003	193 (46%)	539 (59.6%)	<0.001
Tall stature	8 (0.9%)	8 (0.5%)	0.224	5 (1%)	3 (0.4 %)	0.180	3 (0.7%)	5 (0.6%)	0.723
Puberty	78 (8.5%)	217 (13%)	0.001	35 (7%)	48 (6.2%)	0.584	43 (10.2%)	169 (18.7%)	<0.001
Sex development and gender	28 (3%)	39 (2.3%)	0.272	22 (4.4%)	35 (4.5%)	0.902	6 (1.4%)	4 (0.4%)	0.053
Obesity	132 (14.3%)	125 (7.5%)	<0.001	75 (15%)	63 (8.2%)	<0.001	57 (13.6%)	62 (6.9%)	<0.001
Pituitary gland, hypothalamus, and central nervous system	9 (1%)	9 (0.5%)	0.195	7 (1.4%)	6 (0.8%)	0.283	2 (0.5%)	3 (0.3%)	0.689
Thyroid gland	98 (10.7%)	93 (5.6%)	<0.001	38 (7.6%)	32 (4.2%)	0.008	60 (14.3%)	61 (6.7%)	<0.001
Adrenal glands	17 (1.8%)	13 (0.8%)	0.014	9 (1.8%)	8 (1%)	0.249	8 (1.9%)	5 (0.6%)	0.020
Testes and male reproductive tract	35 (3.8%)	64 (3.8%)	0.983	35 (7%)	64 (8.3%)	0.394	-	-	-
Ovaries and female reproductive tract	21 (2.3%)	12 (0.7%)	0.001	-	-	-	21 (5%)	12 (1.3%)	<0.001
Glucose and lipid metabolism	35 (3.8%)	59 (3.5%)	0.710	22 (4.4%)	30 (3.9%)	0.658	13 (3.1%)	29 (3.2%)	0.916
Calcium and phosphate metabolism	32 (3.5%)	25 (1.5%)	0.001	23 (4.6%)	14 (1.8%)	0.004	9 (2.1%)	11 (1.2%)	0.197
Salt and water regulation	6 (0.7%)	1 (0.1%)	0.005	5 (1%)	1 (0.1%)	0.027	1 (0.2%)	0 (0%)	0.142
Syndromes With Endocrine Features	6 (0.7 %)	11 (0.7 %)	0.991	2 (0.4 %)	6 (0.8 %)	0.404	4 (1 %)	5 (0.6 %)	0.409

**Figure 3:**
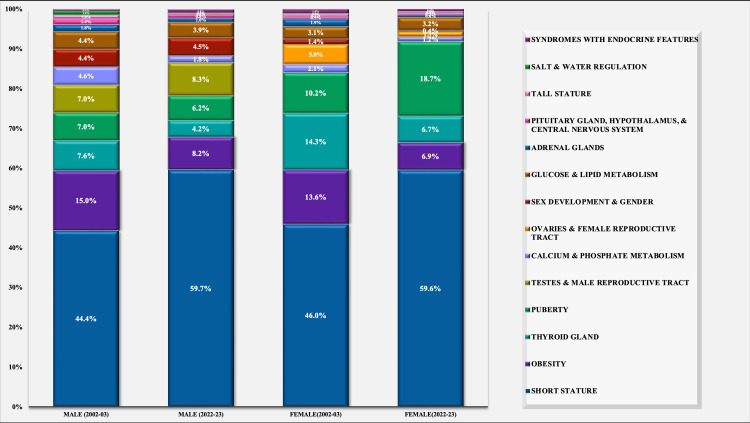
Distribution of referral reasons among males and females as per proportions two decades apart (2002-2003 and 2022-2023)

Short Stature was the most frequent reason for referral overall and across both sexes during the two time periods. Notably, the proportion of referrals for short stature increased significantly, from 45.1% (n=415) in 2002-2003 to 59.6% (n=999) in 2022-2023. While this upward trend was observed in both males and females, statistical significance was evident only in females. The proportion of patients referred for disorders of puberty had increased from 8.5% (n=78) to 13% (n=217) (p<0.001). This increase was predominantly seen in females from 10.2% (n=43) to 18.7% (n=169) (p<0.001), whereas the proportion change in males was not significant (7% to 6.2%). There was a significant rise in the proportion of girls being referred for Precocious puberty over two decades (3.15% (n=13) in 2002-2003 to 8% in 2022-2023 (n=72)). There was a significant reduction in the proportion of patients referred for obesity from 14.3% (n=132) to 7.5% (n=125) (p<0.001). This reduction was significant in both males and females. Similarly, there was a significant reduction in referrals for disorders of the thyroid gland in both sexes and disorders of the thyroid gland and disorders of the ovaries and female reproductive tract in females (p<0.001) (Table 1, Figure 3).

Comparison of referral reasons with final diagnosis

Out of 2595 patients, 5.3% (n=92) had no pathological findings and investigations were within reference range, thus they were labelled as normal. This was commonly seen in patients referred for goiter, adrenal disorders, micropenis, and precocious puberty, where 28% (n=4), 23% (n=6), 16.7% (n=16), and 15% (n=35) patients had no pathological clinical or biochemical findings, respectively. The discrepancy between the final diagnosis and referral reason was predominantly observed in patients referred for micropenis, gynecomastia, and obesity.

Of the 96 patients referred for micropenis, 42.7%(n=41) had buried penis secondary to obesity; of the 54 patients referred for gynecomastia, 22.2% (n=12) had lipomastia due to obesity. Of these 257 patients referred for suspected endogenous or secondary obesity, 85.6% (n=220) had exogenous or primary obesity.

## Discussion

To the best of our knowledge, this is the first study from India where an analysis of the referral pattern of all the pediatric endocrine disorders is described. Overall, the most common reason for referral to a pediatric endocrine clinic in 2022-2023, as well as in 2002-2003, was short stature in both sexes. This was followed by disorders of the testes and male reproductive tract in males and disorders of puberty in females. Obesity was the third most common reason for referral in both sexes. We noticed a substantial increase in the number of referrals to pediatric endocrine clinics from 2002-2003 to 2022-2023. There was a 1.8-fold increase in total referrals over the span of two decades, and the referrals for girls had doubled. The proportion of referrals for short stature and disorders of puberty increased over two decades, particularly in females. A reduction was observed in referrals for obesity and disorders of the thyroid gland in both males and females, and in disorders of the ovaries and female reproductive tract. A discrepancy between the final diagnosis and referral reason was predominantly found in patients referred for micropenis, gynecomastia, and obesity.

We found that more than half of the referrals were for short stature. A similar trend is reported by a study from Italy, where disorders of growth were the most common reason for referral [6]. Among the cases referred for short stature at our center, idiopathic short stature, primary growth failure, and secondary growth failure were the most common subcategories in that order. In these, FSS comprised almost one-fourth of all the cases of short stature. Like our findings, a study from Bangladesh has also reported the most common cause of short stature as FSS [10]. Whereas in a study from Northern India and in another from Pakistan, CDGP was reported as the most common cause of short stature [11,12].

There was an increase in the total number of referrals for short stature over the two decades. Although the increase was observed in both sexes, the rise was more pronounced in females. This rise could be due to increased awareness among parents and physicians about treatment options, improved screening and growth monitoring tools such as manual or electronic plotting of growth charts, efforts and guidelines published by national pediatric societies, parental expectations and societal pressures, early genetic diagnosis of syndromes associated with short stature as well as the modifications in the indication of growth hormone therapy [13,14].

Among disorders of puberty, two-thirds of patients were referred for precocious puberty. Our study identified a significant rise in referrals for precocious puberty in girls between 2002-2003 and 2022-2023. The earlier onset of puberty, influenced by secular trends, increasing prevalence of obesity, lifestyle changes, and increased exposure to endocrine-disrupting chemicals (EDCs) during and after the COVID-19 pandemic, has been reported in multiple countries, including India [15,16]. Similarly, a 20-year nationwide study from Denmark documented a significant increase in the annual incidence of central precocious puberty and normal variants of puberty in girls of Danish origin, with a comparable but less pronounced trend observed in boys [17].

In children referred for obesity, polygenic multifactorial obesity was the most common subcategory. As per a WHO report, the prevalence of obesity among children and adolescents aged 5-19 has risen dramatically from just 2% in 1990 to 8% in 2022 [18]. According to the Indian National and Family Health Survey (NFHS) report, the prevalence of overweight children in India under five years of age has increased from 2.1% (2015-2016) to 3.4% (2019-2021) [19]. A meta-analysis of data over two decades from India has reported a prevalence of childhood obesity of 8.4% [4]. However, we found a significant decline in the number of referrals for obesity over two decades, but there was a rise in referrals for micropenis and gynecomastia. These were found to be manifestations of obesity in half and one-third of total referrals for micropenis and gynecomastia, respectively. Thus, although the referral for obesity appears to have been reduced, when micropenis and gynecomastia are added to the obesity referral, the proportion referred for obesity is 12.4% (n=207). Also, continuing medical education may have improved primary health care providers' knowledge about obesity, that it is primarily a lifestyle disease and not an endocrine disease in most cases.

Hypothyroidism was the most common reason for referral in disorders of the thyroid gland. Most of these referrals were already diagnosed or clinically suspected of hypothyroidism, in contrast to the other studies, where the children were referred for short stature and later found to have hypothyroidism [12,20]. This may reflect a lower threshold for ordering a thyroid function test by the primary physician/pediatrician in children with either obesity or a family history of thyroid disease. The referrals for disorders of the thyroid gland have significantly decreased over the past two decades, suggesting that a substantial proportion of these cases are being effectively diagnosed and managed by primary health care providers.

Among the disorders of the testes and male reproductive tract, disorders of the penis were the most common referral reason, followed by the non-pathological normal variants. However, about one-fifth of patients with disorders of the penis were falsely categorized as micropenis by referring physicians or pediatricians, while the final diagnosis was buried penis, secondary to obesity.

We noticed a substantial increase in the number of referrals to our pediatric endocrine clinic from 2002-2003 to 2022-2023, with a 1.5-fold rise in males and more than a twofold rise in females. A similar trend was reported in an Italian study, where these referrals increased over six years despite a decline in the overall population [8]. The most notable increase was in referrals for disorders of puberty and short stature, with a higher proportion of girls referred for pubertal disorders. No sex bias was identified among children referred for short stature. This suggests that parental concerns regarding growth were similar for both sexes, with no significant differences in health-seeking behavior for short stature observed between boys and girls.

The number of referrals for ovarian and female reproductive tract disorders has significantly declined despite a high prevalence of menstrual disorders among adolescent girls in India [21,22]. This shift is likely due to increased referrals to gynecologists instead of endocrinologists, driven by the greater accessibility to the gynecological specialty, cultural perceptions associating menstrual issues with gynecology, and overlapping expertise.

A discrepancy was observed between the referral reasons and final diagnoses, particularly in patients referred for micropenis, gynecomastia, and obesity. The proportion of obesity cases misclassified as micropenis remained consistent over two decades, inappropriately inflating the initial categorization of disorders of the testes and male reproductive tract. Cases of lipomastia secondary to obesity frequently get misclassified as gynecomastia and hence categorized under disorders of Puberty during initial evaluation. Approximately one-fourth of patients referred for precocious puberty were found to have no pathological findings and were categorized as normal. Additionally, most obesity referrals for suspected endocrine causes were ultimately attributed to inappropriate dietary and lifestyle practices. This highlights the need to spread awareness among primary care pediatricians/physicians about presenting complaints of common disorders, such as obesity, where the patient presents with perceived micropenis or acanthosis, but has simple obesity, which needs attention.

This study provides a comprehensive analysis of referral patterns to a pediatric endocrine clinic in Western India and evaluates the evolving referral practices over the past two decades, highlighting disparities between the perceived and the final diagnoses, offering insights into further education of both primary care providers and specialists.

A potential limitation of this study is its retrospective design. The data largely represent the middle or upper-middle socioeconomic class population, as it is from a hospital catering to this population; the data from a public sector hospital was not taken into consideration. Further, the data are from a single center. However, the main findings on prevalence, change in trend, gender distribution, and discrepancy in referral reasons reported in our study are consistent with other studies in the literature.

## Conclusions

We describe recent status and the evolution of patterns of pediatric endocrine referrals over two decades, revealing a significant shift in the number of referrals and a minor shift in the reasons for referrals. A gap exists in recognizing the symptoms and possible cause at the primary care level. These findings emphasize the importance of education and awareness among both primary care providers and specialists. While this study highlights these gaps, future research may be directed at the reasons that cause change in the referral pattern over time.
